# Nrf2-Mediated Antioxidant Defense and Thyroid Hormone Signaling: A Focus on Cardioprotective Effects

**DOI:** 10.3390/antiox12061177

**Published:** 2023-05-30

**Authors:** Laura Sabatino

**Affiliations:** Institute of Clinical Physiology, National Research Council, Via Moruzzi 1, 56124 Pisa, Italy; laura.sabatino@cnr.it; Tel.: +39-050-315-2659

**Keywords:** thyroid hormones, oxidative stress, antioxidants, Nrf2, cardioprotection

## Abstract

Thyroid hormones (TH) perform a plethora of actions in numerous tissues and induce an overall increase in metabolism, with an augmentation in energy demand and oxygen expenditure. Oxidants are required for normal thyroid-cell proliferation, as well as for the synthesis of the main hormones secreted by the thyroid gland, triiodothyronine (T3) and thyroxine (T4). However, an uncontrolled excess of oxidants can cause oxidative stress, a major trigger in the pathogenesis of a broad spectrum of diseases, including inflammation and cancer. In particular, oxidative stress is implicated in both hypo- and hyper-thyroid diseases. Furthermore, it is important for the TH system to rely on efficient antioxidant defense, to maintain balance, despite sustained tissue exposure to oxidants. One of the main endogenous antioxidant responses is the pathway centered on the nuclear factor erythroid 2-related factor (Nrf2). The aim of the present review is to explore the multiple links between Nrf2-related pathways and various TH-associated conditions. The main aspect of TH signaling is described and the role of Nrf2 in oxidant–antioxidant homeostasis in the TH system is evaluated. Next, the antioxidant function of Nrf2 associated with oxidative stress induced by TH pathological excess is discussed and, subsequently, particular attention is given to the cardioprotective role of TH, which also acts through the mediation of Nrf2. In conclusion, the interaction between Nrf2 and most common natural antioxidant agents in altered states of TH is briefly evaluated.

## 1. Molecular Aspects of Thyroid-Hormone Signaling: An Overview

The thyroid hormones (THs) include the prohormone thyroxine (T4) and the biologically active form triiodothyronine (T3) and regulate a wide range of genes, intervening in many physiological processes, such as cell growth, development, differentiation, and survival [1]. They are synthesized in the thyroid follicles after the iodination of thyroglobulin (Tg) by thyroid peroxidase (TPO) [2].

Largely in the form of T4, THs are released in the circulation, where they are mostly bound to transport proteins and reach the peripheral tissues, where the 5′-monodeiodinases (DIO1 and DIO2) catalyze T4 to T3 activation [2]. A third monodeiodinase (DIO3) has been described in the cells and mediates T4 conversion to metabolically inactive reverse T3 (rT3) [2].

**Figure 1:**
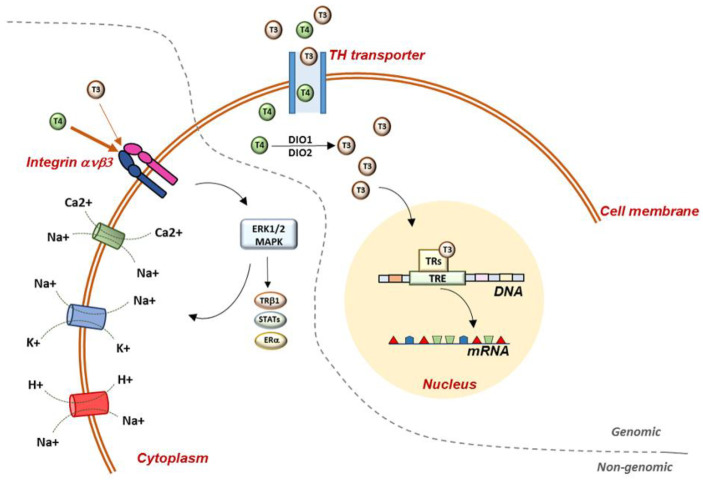
Representation of genomic and non-genomic actions of TH in the cell. Genomic actions begin at the plasma membrane and THs enter the cell through specific TH transporters. Once in the cell, T4 is converted to T3 by D1 and D2 deiodinases and T3 enters the nucleus, where it binds to specific receptors, which mediate the interaction with the DNA. Non-genomic mechanisms require the mediation of integrin ανβ3, which has a higher binding affinity for T4 than T3. Once in the cell, THs activate several MAPK-mediated signaling pathways. At the plasma-membrane level, TH regulate glucose transporter, Na^+^/K^+^-ATPase, Na^+^/H^+^-exchanger, Ca^2+^-ATPase, and the Na+-sensitive amino-acid transporter. TH: thyroid hormones; T3: triiodothyronine; T4: thyroxine; TRs: thyroid-hormone receptors; TRE: thyroid-responsive elements; DIO1: deiodinase 1; DIO2: deiodinase 2; STATs: signal transducer and activator of transcription 1α and 3; ERα: estrogen receptor α.

The signaling of TH in the target cells is highly complex and finely regulated [3]. Transporters of TH mediate the uptake of TH and, once inside the cell, TH can mediate genomic effects in the nucleus, binding to specific receptors (TRs), which directly interact with responding elements (TREs) in the target promoters, thus regulating the transcription of specific genes (Figure 1) [4]. Two genes encoding TRs have been described, THRA and THRB, codifying for TRα and TRβ, respectively, and these two isoforms are differently expressed during embryonic development and in adult life [1,5].

Furthermore, TH actions can also be exerted by the so-called non-genomic mechanisms, which occur in a short time (from seconds to minutes) and do not require the direct interaction of TH with TRs and DNA, but act trough intracellular signaling pathways, which indirectly regulate gene transcription [6]. Most of these effects, observed in different tissues, begin with TH interactions with receptors located in the plasma membrane, mitochondria, or cytoplasm [7,8,9]. The receptors involved in non-classical actions may or may not have structural homologies with TRs, and some actions that initiate at the plasma membrane may also regulate the fate and function of nuclear TRs [6].

In the plasma membrane, the heterodimer protein integrin ανβ3 has been demonstrated to be of central importance in the mediation of TH effects on cell angiogenesis and proliferation; in fact, high concentrations of integrin are detectable on vascular and tumor cells. Integrin ανβ3 interacts with many structural proteins in the extracellular matrix, playing a crucial role in transducing important signals either from the outside into the cells or from the intracellular to the extracellular compartment [9]. The main intracellular signaling cascade triggered by TH-integrin ανβ3 interaction is the mitogen-activated protein kinase (MAPK; ERK1/2) via phospholipase C (PLC) and protein kinase Cα (PKCα). The TH-activated MAPK mediates the serine phosphorylation of several nuclear trans-activator proteins, such as the signal transducer and activator of transcription 1α and 3 (STAT1α and STAT3), TRβ1, estrogen receptor α (ERα), etc. [10,11]. Furthermore, at the plasma-membrane level, TH effects have been associated with the regulation of essential membrane-transport systems, such as glucose transporter, Na^+^/K^+^ -ATPase, Na^+^/H^+^-exchanger, Ca^2+^-ATPase, and the Na^+^-sensitive amino-acid transporter (Figure 1) [12,13,14].

Moreover, acting on a cytoplasmic truncated form of nuclear TRα1 (TRΔα1), TH can regulate dynamic and structural changes in the cellular architecture, through the conversion of soluble to fibrous actin, which is important for cells’ motility and interaction with the environment (for example, in glia and neurons). In vitro studies showed that the ability of astrocytes to adhere to the culture dish was associated with the presence of TH, and that the deprivation of TH in the culture medium induces the loss of the major actin filaments in the cells. It was demonstrated that, upon the administration of TH to the culture medium, this effect can be rapidly reversed in a few minutes by the mediation of truncated delta TRs, TRΔα1 and TRΔα2 [15].

Of the principal extra-nuclear activities, the regulatory action of TH on mitochondria is of central importance in cell metabolism and requires the presence of truncated TRα isoforms. More specifically, truncated forms of nuclear TRα1, with molecular weights of 43 kDa (p43) and 28 kDa (p28), have been described in the matrix and the inner membranes of mitochondria, respectively [16,17,18]. These forms lack of DNA-binding domain of TRα1, and the binding affinity of p28 for T3 is higher than that of p43 [15]. Furthermore, both receptors are targeted to mitochondria, but only p28 was demonstrated to enter the organelle in a T3-dependent way [17]. In the last decade, many other forms of nuclear-receptor superfamily have been described in the mitochondria, suggesting that the physiological importance of the interaction between TH and the organelles may be more complex than initially thought [18].

It is well known that TRs and steroid receptors have similar structural characteristics and conserved domains, and that they play a major role in the mediation of TH and steroid-hormone regulatory activity in the mitochondrial enzymes of oxidative phosphorylation (OXPHOS) [19]. It is believed that TH and steroid hormones can coordinate OXPHOS nuclear and mitochondrial gene expression and protein biosynthesis, in both normal and pathological contexts, in order to regulate energy metabolism [20]. The finding in the mitochondrial genome of sequences similar to nuclear hormone-response elements (HREs) and the presence of TRs and steroid receptors in mitochondria suggest the possibility of parallel hormonal regulation at the nuclear and mitochondrial level [21]. Interestingly, in the nucleus, the TH and steroid-hormone activation of OXPHOS genes can occur through both direct and indirect interaction with HRE-containing genes encoding for specific transcription factors, such as nuclear respiratory factors 1 and 2 (NRF1 and NRF2) and peroxisome proliferator-activated receptor γ coactivator 1α (PGC-1 α), which, in turn, can induce genes encoding mitochondrial transcription factors, such as TFAM, TFB1M,s and TFB2M, which activate mitochondrial OXPHOS gene expression [21].

## 2. Oxidant–Antioxidant Homeostasis: Nrf2 and Thyroid Protection

Oxidants are required for regular activities in cells and are continuously produced by endogenous processes or obtained from exogenous sources. The basal level of oxidants is maintained by active oxygen scavenging through enzymatic superoxide dismutase (SOD), catalase (CAT), glutathione peroxidase (GPx), and glutathione reductase (GR), as well as non-enzymatic antioxidant molecules, such as reduced glutathione (GSH) [22].

Several organelles, including mitochondria, the endoplasmic reticulum (ER), and peroxisomes, as well as enzymatic systems, such as xanthine oxidase, lipoxygenase, and nitric oxide synthase, are important sources of oxidants in mammalian cells [23]. In normal cells, oxidants are mainly generated by mitochondrial oxidative phosphorylation, and moderate amounts of oxidants have positive effects as regulators of inflammation, immune activity, and stress response, as protectors against invading harmful pathogens and as mediators of healing and repairing processes [22]. In addition to mitochondria, other organelles, such as the endoplasmic reticulum and peroxisomes, can produce oxidants, and their relative contributions vary according to the cell type [23]. Oxidative stress is an effect of a redox imbalance between oxidants and antioxidant defense. It can be induced by the excessive production of oxidants and/or reduced antioxidant capacity, thus provoking molecular damage [24]. Furthermore, oxidants produced in different cellular compartments determine a positive feedback circuit, supporting pathological conditions associated with oxidative stress [25,26]. 

More specifically, in the thyrocytes, H_2_O_2_ is the primary oxidative agent required by the TPO enzyme for regular hormonogenesis; hence, oxidants are continuously produced, even in physiological conditions [27]. However, since an uncontrolled excess of oxidants can rapidly cause oxidative stress, follicular cells have to guarantee the presence of efficient protective mechanisms. Recently, the antioxidant pathway centered on the nuclear factor erythroid 2-related transcription factor 2 (Nrf2) and its cytoplasmic inhibitor, Kelch-like ECH-associated protein (Keap1) has gained increasing relevance as an efficient antioxidant system in the thyroid [28,29].

**Figure 2:**
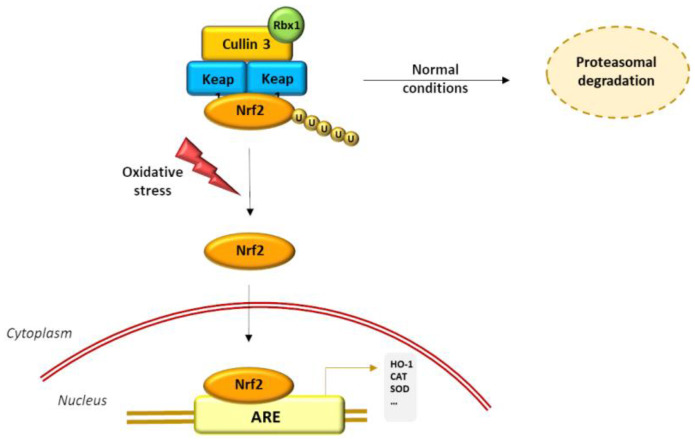
Representation of Nrf2–ARE pathway activation by oxidants. In normal conditions, Keap1 acts as an adaptor targeting Nrf2 and inducing proteasomal degradation. In presence of excess oxidant stimuli, Keap1’s stabilizing function is inactivated and Nrf2 accumulates in the nucleus. At the nuclear level, Nrf2 acts as a transcription factor interacting with the ARE sequences in the promoters of numerous target genes encoding antioxidant enzymes and other cytoprotective molecules.

In normal conditions, Keap1 acts as an adaptor targeting Nrf2 for poly-ubiquination by Cullin 3-based ubiquitin E3 ligase (Cul3-Rbx1) and proteasomal degradation. In the presence of redox-disrupting stimuli, Keap1 thiol groups react with oxidants, such as H_2_O_2_, leading to the inactivation of the Keap1 stabilizing function, inducing the impairment of Nrf2 poly-ubiquination and the accumulation of Nrf2 in the nucleus. At the nuclear level, Nrf2 acts as a transcription factor, interacting with the so-called antioxidant response elements (AREs) in the promoters of numerous target genes, encoding antioxidant enzymes and other cytoprotective molecules (Figure 2) [30]. Furthermore, Nrf2 is believed to control the basal and inducible expression of over 1000 genes involved in antioxidant defense, detoxification, inflammatory response, and proteasomal and autophagic degradation and metabolism. The role of Nrf2 was extensively studied in Nrf2 knockout mice, in which the expression level of antioxidant and cytoprotective genes decreased, whereas a higher level of oxidative damage was augmented [31]. Although Nrf2 is ubiquitously expressed, its role as a multiple-organ protector is not only due to the regulation of ubiquitous cytoprotective genes, but also to the regulation of tissue-specific genes involved in highly specialized functions in different tissues [32].

While a minimal oxidative load is required for normal thyroid-gland function, antioxidant protection is activated when oxidative stress occurs, in conditions of iodine overload; Nrf2, in association with its cytoplasmic inhibitor Keap1, is considered the main mediator of the antioxidant response [33]. Experimental studies evidenced that Nrf2 promotes antioxidant defense in the thyroid gland by stimulating the expression of cytoprotective molecules, such as GPx2, GR1, thioredoxin 1 (TXN1), thioredoxin reductase 1 (TXNRD1), sulfiredoxin 1 (SRXN1), and NAD(P)H quinone dehydrogenase (NQO1), which are known to have determining roles in regular thyroid activity [34,35,36]. In a study on a Nrf2-KO mouse model, it was shown that the cytoprotective activity was dramatically lost after iodine overload, whereas in wild-type mice, despite the excessive iodine exposure, no augmented protein or lipid levels were observed, suggesting that Nrf2-dependent antioxidant machinery was efficiently activated to neutralize oxidative-stress onset [33]. Furthermore, in the same study, it was observed that Nrf2, in addition to maintaining thyroid homeostasis, plays an important role in the regulation of Tg synthesis through the direct regulation of ARE sequences present in the Tg gene, both in rodents and in humans. Interestingly, the fact that in Nrf2-KO mice, a relevant increase in iodinated Tg was observed indicates that Nrf2 is involved in the regulation of both the synthesis and the iodination of Tg [33]. 

**Figure 3:**
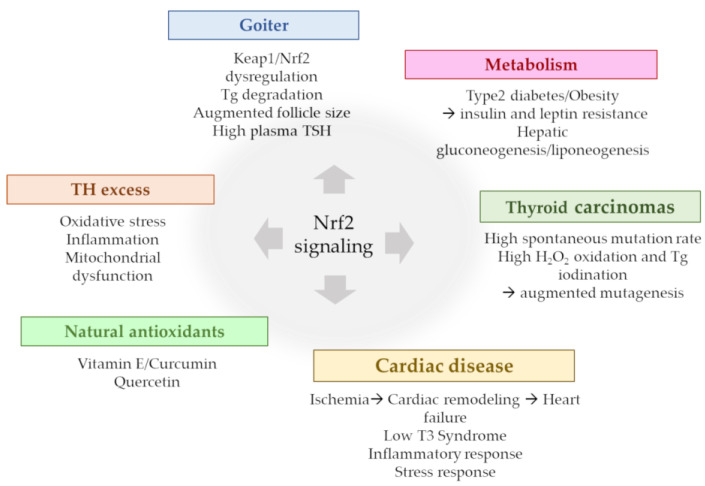
Nrf2 signaling in different pathological contexts.

The main Nrf2 signaling aspects in thyroidal pathological contexts, discussed in the present review, are summarized in Figure 3. 

Some reports on mice and humans showed the involvement of the Keap1/Nrf2 system in goiter formation and that the gene-based over-activation of Nrf2 in the thyroid can induce goiter formation with variable phenotypic characteristics [33,37]. Germline mutations of Keap1 are very rare and usually associated with non-toxic multinodular goiters, characterized by the nodular enlargement of the thyroid without thyroid dysfunction or inflammation [38]. It is reasonable to assume that the germline Keap1 mutation may determine Nrf2 activation in the thyroid, as in all other tissues; however, no diseases other than goiters were described in the patient affected, and this intriguing aspect needs to be investigated further [32]. The most frequently used experimental model for Nrf2-pathway activation is the Keap1 knockdown (Keap1^KD^) mouse, also referred as Keap1 hypomorphic, which has very low expression levels of the Keap1 gene [39]. Keap1^KD^ mice have enlarged goiters, with dilated follicles, the absence of nodules and hyperplasia, and decreased plasma levels of T4, which are normalized in adult life by the activity of stimulating hormone (TSH), the pituitary hormone that stimulates follicular thyroid-cell growth and function [40]. Furthermore, the finding in Keap1^KD^-mouse thyroids of high concentrations of Tg-degrading cathepsin enzymes indicates that the lysosomal degradation of Tg may offer important support for the pathogenesis of goiters in Keap1^KD^ mice [28,40]. 

The Keap1^KD^ mouse model was also used in studies of metabolic diseases, especially in the context of type 2 diabetes and obesity, where, in addition to its protective role against oxidative-stress damage, Nrf2 can interact with pathways not directly associated with cytoprotection, such as in the hypothalamus, where Nrf2 improves insulin and leptin resistance, preventing the progression of diabetes mellitus [41], or in the liver, where Nrf2 has been described as a potential repressor of hepatic gluconeogenesis and lipogenesis [42]. Unfortunately, no data on thyroids were reported in these studies, which might be an interesting starting point for future studies.

The signaling of Nrf2 was also found to be activated in thyroid carcinomas, where it exerts a dual role, since, in addition to conferring protection against oxidative stress, it also promotes drug resistance to malignant cells [32,43]. Thyroid tumors are quite frequent in the population and the spontaneous mutation rate (preferentially single-base modifications) in the thyroid is higher than in other tissues. Furthermore, the presence of H_2_O_2_ for iodine oxidation and Tg iodination might account for the high mutagenesis rate in the thyroid [44]. In the attempt to better define the molecular mechanisms leading to Nrf2 activation in thyroid cancer, several Nrf2-gain-of-function and Keap1-loss-of-function somatic mutations were described in many human cancers in different tissues, including the liver, kidneys, lungs, and others [43,45]. The Nrf2 appears to be able to function not only as a tumor suppressor but also as an oncogene. In fact, while Nrf2 initially acts as a cancer-preventive factor, protecting cells from carcinogens and oxidative stress, the persistent activation of Nrf2 activates its oncogene properties and reduces radiotherapy- and chemotherapy-induced cytotoxicity, enhancing drug resistance in cancer cells [46].

Therefore, the inhibition of Nrf2 signaling is increasingly considered a potential target to overcome drug resistance and provides a novel strategy through which to increase the efficacy of traditional treatments.

## 3. Thyroid Hormone Excess, Oxidative Stress, and Nrf2 Activation

Diseases of THs are strongly associated with oxidative stress, and while, on one hand, oxidants interfere with the synthesis, activity, and metabolism of hormones, the reverse condition is also possible, and TH can regulate the antioxidant levels in cells. 

Depending on the tissue demand, in normal conditions, a baseline level of oxidants is necessary to preserve cell homeostasis, and this number of oxidants is generally low in most tissues. When the oxidants exceed the ability of the cells to remove the oxidant surplus, oxidative stress arises. Thus, the role of oxidants in cells depends mainly on their initial concentrations, which determine the downstream cellular responses. 

The THs are key regulators of cellular metabolism, and several studies found that in hyperthyroidism, the augmented metabolic demand promotes the synthesis of chemical energy by mitochondrial oxidation-reduction reactions, thus increasing the oxidant levels in the cell and inducing lower antioxidant ability [47,48].

The available data indicate that TH administration increases H_2_O_2_ generation by mitochondria in rat tissues, and this event is often associated with increased rates of oxygen consumption in target tissues, such as the liver, kidneys, heart, and skeletal muscles, where the need for metabolic capacity is higher [49,50]. Variability in the antioxidant response leading to an imbalance in oxidant clearance was observed in the tissues of hyperthyroid-induced animals, and further variations were appreciable, according to the age and the characteristics of the animals undergoing TH treatment [51]. 

Hyperthyroidism and thyrotoxicosis have been associated with the activation of Nrf2 signaling in TH target tissues. More specifically, in rat livers, T3 administration led to a rapid and transient cytosol-to-nuclear translocation of Nrf2, and it was hypothesized that the increase in oxidative status induced by T3 administration may inactivate Keap1-mediated ubiquination/degradation and expand Nrf2 nuclear-pool availability [52]. 

Several studies hypothesized that Nrf2 activation is triggered by mitogen-activated protein kinases (MAPKs) produced by T3-induced oxidants; however, the exact role of MAPKs and the underlying molecular mechanism remain poorly defined [52]. On the other hand, some other studies evaluated the hypothesis that the direct phosphorylation of Nrf2 by MAPKs contributes little to the modulation of Nrf2 activity and suggested that MAPKs mainly regulate the Nrf2 signaling pathway through indirect mechanisms [53]. 

In the last decades, several studies have provided new approaches to detailing the interactions between the TH system and mitochondrial compartments and to elucidating the effects of TH on electron-transport complexes and the existing relationship with oxidative metabolism [54]. Recently, it was demonstrated that respiratory complexes are organized in higher-order structures, called supercomplexes, which guarantee the major stabilization of the assembly and better control over oxidant production in the electron-transport chain, thanks to the better accessibility of substrates necessary for enzymatic reactions [55,56]. Moreover, the discovery of supercomplexes represents an important step forward in the study of the functional and structural properties of the mitochondrial respiratory chain, even though their functional advantages and their possible pathophysiological involvement in TH disease are far from being fully understood.

In an experimental model of hyperthyroidism, it was found that more than 58% of mitochondria were swollen, and that their cristae were radially oriented towards the center of organelles [57]. Alterations in mitochondrial morphology can slightly reduce the efficiency of phosphorylation, whereas the TH-induced increase in mitochondrial respiratory complexes explains the increase in respiratory rate [58].

In normal conditions, Nrf2 affects the mitochondrial membrane potential, fatty-acid oxidation, the availability of substrates for respiration (NADH and FADH2/succinate), and ATP synthesis. In conditions of stress, Nrf2 activation counteracts oxidant production in mitochondria via the transcriptional upregulation of uncoupling protein 3 and influences mitochondrial biogenesis by maintaining adequate levels of NRF1 and PGC-1α, as well as by promoting purine-nucleotide biosynthesis in rapidly growing cells [59].

The Nrf2 plays an important role in the maintenance of mitochondrial homeostasis and structural integrity. This is especially true in conditions of oxidative, electrophilic, and inflammatory stress, when the request for cytoprotective responses is crucial for the survival of the cell and the organism. The effects on mitochondria are among the principal protective mechanisms mediated by Nrf2. Diseases of the THs, analogously to many other pathological conditions, are characterized by oxidative stress, inflammation, and mitochondrial dysfunction as essential components of their pathogenesis. Therefore, Nrf2’s possible involvement holds promise for disease prevention and treatment.

## 4. Thyroid Hormones and Their Antioxidant Role in Cardioprotection: Nrf2 Mediation

Experimental studies showed the negative effects of TH-altered metabolism on cardiac function, cell protection, and mitochondrial function, whereas the reversibility of these conditions restores the euthyroid state, suggesting that TH exert an important cardioprotective role [60].

Oxidative stress is a determining factor in the pathological progression of cardiac diseases, and excess of oxygen species may occur when oxygen supply is limited, such as during cardiac ischemia. In these conditions, oxidants can provoke irreversible damage by oxidation-membrane phospholipids, proteins, and DNA [61]. Subsequently, the heart reacts with a remodeling process that starts as a compensatory event characterized by the hypertrophy of surviving myocytes and the fibrosis of non-myocyte components, but soon involves the activation of the neuroendocrine and inflammatory systems and leads to decompensation and heart failure [62]. In particular, the progression to heart failure is associated with a progressive compromise of mitochondrial respiratory activity and a reduction in its capacity to produce ATP, which, in turn, leads to secondary dysregulation and altered Ca^2+^ handling and energy deficiency [63].

In both clinical settings and experimental studies of acute myocardial infarction, the reduction in circulating T3 levels (low-T3 syndrome) is one of the principal alterations observed and correlates with intense pro-inflammatory and stress responses [64]. The low-T3 state induces several important molecular, biochemical, and histological changes in the myocardium [65] and, for a long time, low T3 has been considered part of a beneficial adaptive mechanism aiming to reduce cardiac energy expenditure. However, clinical and experimental data demonstrated that low T3 is a strong prognostic predictor of short-term and long-term mortality [66,67], and that constant and low-level T3 administration allow the normalization of the hormone in the serum, attenuate myocardial damage, reduce remodeling, and prevent oxidative stress, with the final effect of improving cardiac function [68,69].

Many Nrf2-regulated enzymes are involved in the pathogenesis of cardiovascular diseases and may act as specific markers of the progression towards heart failure. These genes include antioxidant-related genes [70], stress-response genes [71], and genes limiting the inflammatory processes and conferring protection against ischemia/reperfusion events [72].

Coronary artery disease and ischemic heart disease are the most prevalent causes of mortality worldwide, and post-myocardial infarction hypertrophy, fibrosis, and apoptosis are the major events driving the progression towards heart failure. Coronary interventions and revascularization initially provide benefits after acute myocardial infarction; however, ischemia/reperfusion injury occurring during revascularization may worsen general cardiac conditions due to oxidant formation and inflammatory infiltration [72,73,74,75]. In this context, Nrf2 has been demonstrated to play a central role in cardiac protection, through the regulation of a broad spectrum of target genes [76,77]. 

**Figure 4:**
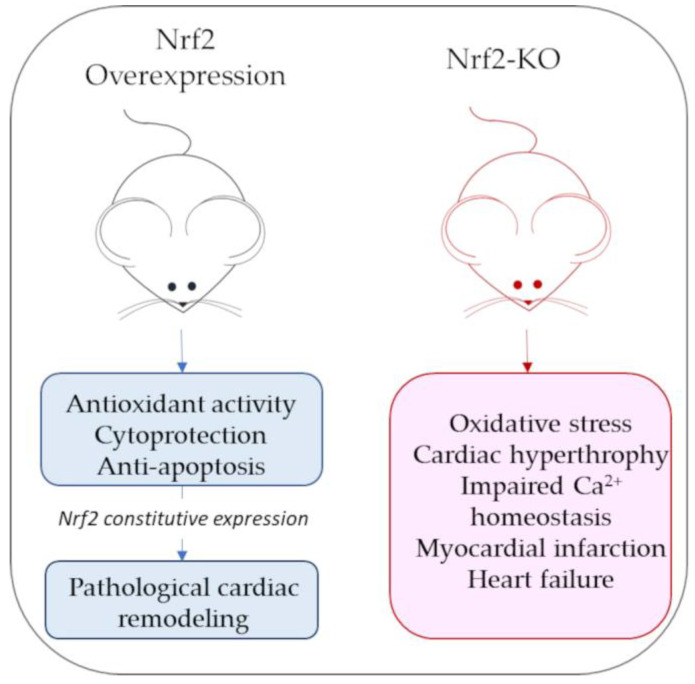
Schematic representation of main cardiac phenotypes associated with Nrf2 overexpression or lack of expression (Nrf-KO mice).

Mouse models of Nrf2 overexpression or Nrf2 knockouts have been widely used to characterize Nrf2’s role in cardiac pathological contexts. In mice with constitutively active Nrf2 cardiac overexpression, beyond the increased expression of antioxidant genes, some hypertrophic genes (i.e., genes for natriuretic peptides A and B) are also stimulated, increasing the risk of developing pathological cardiac remodeling [78]. By contrast, Nrf2-KO mice have a marked exposure to oxidative insult and oxidative-stress-associated pathologies [79]. Although some antioxidant gene expression is still appreciable in Nrf2-KO mice, it is not sufficient to compensate for oxidative stress and cardiac hypertrophy due to acute exercise stress, leading to cardiac dysfunction [79,80]. Figure 4 reports a schematic representation of the main cardiac phenotypes associated with Nrf2 over expression or lack of expression.

Several antioxidant agents exert their protective effects on ischemia/reperfusion injury through the induction of Nrf2-regulated pathways [81,82,83,84,85]. Some examples of Nrf2’s effects in myocardial ischemia/reperfusion experimental models are reported in Table 1. 

The Nrf2 affects cell survival through some mediators, such as the anti-apoptotic proteins Bcl-2 and the heme oxygenase-1 (HO-1), a stress protein with antioxidant, anti-apoptotic, anti-thrombotic, and anti-inflammatory properties [86], and it is therefore considered a reliable marker of oxidative stress [87]. During ischemia/reperfusion, Nrf2’s dissociation from Keap1 is encouraged and Nrf2 translocation to the cardiomyocyte nucleus increases, thus increasing antioxidant responses [88]. The stimulation of Nrf2 in cardioprotection is associated with the activation of the pro-survival pathway phosphoinositide 3-kinase (PI3K)/Akt kinase, which is considered a key factor in many aspects of cardiac physiology, such as cell survival, contractility, and electrophysiology [89]. Moreover, the PI3K/Akt pathway is considered to be involved in T3 protection against ischemic injury, both in vivo and in vitro. In fact, in H_2_O_2_-treated cardiomyocytes, pre-treatment with T3 stimulates PI3K and Akt signaling through their phosphorylation [88,89], and in a mouse experimental model, TH-replacement therapy restores myocardial function after ischemia/reperfusion injury [90]. Th levels of Nrf2 increase in response to T3 treatment, suggesting the pivotal role of this factor in the mediation of T3’s protective function in cardiomyocytes [88]. Moreover, HO-1, which is regulated by Nrf2, is also augmented after T3 treatment in vitro, supporting resistance to oxidative stress and mitochondrial biogenesis [59].

## 5. Natural Antioxidants, TH Signaling, and Nrf2 Mediation

Several conditions and chemical substances can interfere with thyroid function and affect the secretion of thyroid hormones and their availability to target tissues. Altered TH levels can cause relevant changes in the ratio of antioxidant enzymes leading to imbalances in the clearance of oxidants, leading to the deterioration of cellular proteins, lipids, and DNA.

In recent years, many efforts have been made to individuate antioxidant molecules commonly present in nature as therapeutic agents to counter the effects of excessive TH-induced oxidant production [91]. In the present paper, the effects of some natural substances and their modulation of Nrf2 signaling in the antioxidant response to altered TH levels will be discussed. 

Vitamin E and curcumin, low-molecular-mass antioxidants, are considered among the most effective natural protective agents against the oxidative stress occurring in hyperthyroidism. They both have a potent oxidant-scavenging activity, but their methods of action are quite different [91]. Vitamin E is lipid-soluble and, once incorporated in the membrane bilayer, interferes with the synthesis of lipid peroxides and carbonyl groups, acting as a membrane stabilizer and limiting lipid-chain peroxidation [92]. Curcumin is a phenolic compound isolated from the rhizome of turmeric (Curcuma longa), which is commonly employed as a spice and food colorant and used in traditional Indian and Chinese medicine for its antioxidant, anti-inflammatory, and anti-carcinogenic properties [93]. Curcumin exerts scavenging effects, directly quenching free radicals or intervening in the oxidative cascade, preventing oxidant formation [94]. Furthermore, it was found that curcumin may inhibit the oxidation of low-density lipoproteins (LDL), thus playing an important role in cardiovascular protection [95]. In addition, curcumin may induce conformational changes in sarcoplasmic reticulum Ca^2+^-ATPase (SERCA), preventing the enzyme from interacting with ATP and blocking Ca^2+^ from entering the sarcoplasmic reticulum [96].

A recent study on rat hearts supported the idea that Nrf2 may be activated after vitamin E/curcumin administration and that it neutralizes altered TH-induced oxidative stress [91]. In oxidant-mediated oxidative damage, the regulation of antioxidant enzymes depends on the TH levels. Hyperthyroidism upregulates SOD and GR and downregulates CAT and GPx, whereas all enzymes are downregulated in hypothyroidism. However, in experimental hyper- and hypo-thyroid animals, in response to vitamin E and curcumin administration, a different regulation of antioxidant enzymes was described. Curcumin alone ameliorated SOD and CAT activities in both TH-altered states, whereas vitamin E alone stabilized SOD and CAT only in hyperthyroid conditions, and no response was observed in the hypothyroid rats. Interestingly, the combined administration of the two compounds normalized the GPx and GR activities compared to the administration of each compound alone. In this study, the optimal in silico interaction observed between vitamin E and curcumin with Keap1 factor strongly suggested that the antioxidant effects of both compounds were mediated by the Keap1-Nrf2 system, Nrf2 release to the nucleus, and ARE-sequence activation [91].

Quercetin is also a flavonoid found in some vegetables and fruits, and it showed antioxidant, anti-inflammatory. and anti-proliferative properties, as well as the ability to suppress lipid peroxidation [97]. In experimental models of hyperthyroidism, it was demonstrated that the administration of quercetin can protect liver functions from oxidative stress [97], and that this effect is mediated by Nrf2 activation and subsequent increases in HO-1 and NQO-1 production [98,99]. Recently, it was observed that quercetin may downregulate the gene expression of thyroid-restricted genes (sodium/iodide symporter, thyroid peroxidase, Tg, and the thyrotropin receptor), indicating that this compound has important inhibitory effects on TH metabolism [100]. However, quercetin might carry a potential toxicity risk if it is administered in excess, since it can inhibit thyroid-cell growth and iodide uptake, thereby negatively interfering with thyroid function. In general, caution should always be exercised in drawing conclusions as to the potential beneficial (or harmful) effects of natural compounds on the metabolism of TH, and a careful toxicological evaluation should be performed in view of their use for therapeutic purposes.

## 6. Conclusions

In summary, Nrf2 can be considered to play a key role in cellular homeostasis, with important regulatory effects on a large battery of genes with cytoprotective actions, exerted in response to different stimuli, such as oxidative stress, inflammation, cell growth, and energy supply. Emerging evidence indicates that Nrf2 plays a critical role in restoring redox balance and metabolic homeostasis in conditions of TH alterations. Ongoing research, including studies on cell cultures and experimental animal models, provides a better understanding of Nrf2 signaling in normal and pathological settings in which TH are involved. While the beneficial role of Nrf2 is well defined, less established is how the regulation of this pathway can be successfully used for disease prevention. First, this difficulty must be ascribed to the fact that developments in research continuously identify the wide range of pathological contexts in which Nrf2 plays a role. Second, the dual function of Nrf2 in disease underlines the need to accurately evaluate the clinical context in developing the most suitable and targeted Nrf2-based therapy.

## Figures and Tables

**Table 1:** Examples of experimental approaches in the study of Nrf2-mediated cardioprotection.

I/R Model	Nrf2 Activation Effects	Downstream Targets	Ref.
Nrf2-KO, C57BL/6J mice	Attenuation of MI size, decreased cardiomyocyte apoptosis	GPx, HO-1, NQO1, TXN1, CYBRD, ALDOSE REDUCTASE	[77]
Fh1-KO mice	Cardioprotection	HO-1, NQO1, MTHFD2, GSTA1	[82]
Sprague-Dawley rats (LA pretreatment)	Reduction of cardiomyocyte necrosis, apoptosis and inflammation	HO-1, GST, SOD, NADPH-regenerating enzymes	[81]
Nrf2-KO, C57BL/6J mice (PGD2 pretreatment)	Cardioprotection	GCLC, GR, G6PD, HO-1, SLC7A11, GSTA2	[83]
C57BL/6J mice (Bortezomib pretreatment)	Redox homeostasis, preservation of cardiac systolic function, reduction of MI size	HO-1, GSH, SOD1, CAT	[84]

GPx: glutathione peroxidase; HO-1: heme oxygenase-1; NQO1: NAD(P)H quinone dehydrogenase; TXN1: thioredoxin 1; CYBRD: Cytochrome b reductase; Fh1: fumarate hydratase 1; MTHFD2: Methylenetetrahydrofolate dehydrogenase (NADP+ dependent) 2; GSTA: glutathione-S transferase; LA: a-lipoic acid; SOD: superoxide dismutase; PGD2: prostaglandin D2; GCLC: glutam Fh1: ate-cysteine ligase catalytic; SLC7A11: solute carrier family 7 member 11; GSH: reduced glutathione; CAT: catalase.

## Data Availability

Not applicable.
